# NGMA-1. Quantification of IDH mutant alleles predicts outcome in diffuse gliomas

**DOI:** 10.1093/noajnl/vdab070.016

**Published:** 2021-07-05

**Authors:** Mathew Voisin, Gelareh Zadeh

**Affiliations:** University of Toronto, Toronto, Canada

## Abstract

**Background:**

IDH mutation is the main factor used in the prognostication of diffuse gliomas, however within IDH mutated gliomas there still remains a high variability in both tumor progression and overall survival.^1^ Digital droplet polymerase chain reaction (ddPCR) is one of the latest molecular amplification techniques that offers high precision in addition to the ability of absolute quantification of mutant allele copies.^2^

**Methods:**

A total of 102 IDH mutant diffuse glioma tumor samples ranging from WHO grade 2 to 4 were collected. This cohort includes a total of 45 paired samples collected at two distinct surgical timepoints: initial and recurrent. All samples underwent DNA extraction. A total of 5 ng of tumor DNA from each sample was analyzed using ddPCR for the detection and quantification of IDH1 R132H mutant alleles. Sanger sequencing was performed on all samples as a gold standard.

**Results:**

ddPCR was highly sensitive (100%) and specific (99%) for the detection of IDH mutations. Initial tumor samples with a high number of IDH mutant copies split by median demonstrated decreased overall survival (p = 0.04) and shorter progression free survival (p = 0.024). The number of IDH mutant copies was independent of WHO grade (p = 0.6) and 1p19q codeletion status (p = 0.86). Tumor pairs that had IDH mutant copies increase at recurrence were trending but not significantly related to a decrease in remaining survival (p = 0.1).

**Conclusions:**

ddPCR is a highly sensitive and specific method of detecting IDH mutations in diffuse gliomas. The number of IDH mutant copies in tumors at initial surgery can serve as an independent prognostic factor to help guide future treatment and follow-up.

